# Addition of plant-growth-promoting *Bacillus subtilis* PTS-394 on tomato rhizosphere has no durable impact on composition of root microbiome

**DOI:** 10.1186/s12866-017-1039-x

**Published:** 2017-06-05

**Authors:** Junqing Qiao, Xiang Yu, Xuejie Liang, Yongfeng Liu, Rainer Borriss, Youzhou Liu

**Affiliations:** 1 Institute of Plant Protection, Jiangsu Academy of Agricultural Sciences, Nanjing, Jiangsu province 210014 China; 2 Suqian institute, Jiangsu Academy of Agricultural Sciences, Suqian, Jiangsu province 223831 China; 3 Institut für Agrarwissenschaften/Phytomedizin, Humboldt Universität zu Berlin, 14195 Berlin, Germany; 4 Nord Reet UG, 17489 Greifswald, Germany

**Keywords:** *Bacillus subtilis* PTS-394, Phylogenomics analysis, Root colonization, Rhizosphere Microbiota community, Roche 454 Pyrosequencing

## Abstract

**Background:**

Representatives of the genus *Bacillus* are increasingly used in agriculture to promote plant growth and to protect against plant pathogens. Unfortunately, hitherto the impact of *Bacillus* inoculants on the indigenous plant microbiota has been investigated exclusively for the species *Bacillus amyloliquefaciens* and was limited to prokaryotes, whilst eukaryotic member of this community, e.g. fungi, were not considered.

**Results:**

The root-colonizing *Bacillus subtilis* PTS-394 supported growth of tomato plants and suppressed soil-borne diseases. Roche 454 pyrosequencing revealed that PTS-394 has only a transient impact on the microbiota community of the tomato rhizosphere. The impact on eukaryota could last up to 14 days, while that on bacterial communities lasted for 3 days only.

**Conclusions:**

Ecological adaptation and microbial community-preserving capacity are important criteria when assessing suitability of bio-inoculants for commercial development. As shown here, *B. subtilis* PTS-394 is acting as an environmentally compatible plant protective agent without permanent effects on rhizosphere microbial community.

**Electronic supplementary material:**

The online version of this article (doi:10.1186/s12866-017-1039-x) contains supplementary material, which is available to authorized users.

## Background

Plant growth-promoting rhizobacteria (PGPR) have potential as biocontrol agents that could replace chemical pesticides thereby reducing undesired chemical remnants in agriculture. Plant growth promotion, suppression of plant diseases and rhizosphere competence (colonization and survival on plant roots) by PGPR, especially bacilli, have been considered as critical requisites for the development of commercial products [1, 2]. To be effective, PGPRs must establish and maintain a sufficient population in the rhizosphere [3]. It is reported that the robust colonization of plant roots by beneficial microbes directly contributes to the effective biocontrol of soil-borne pathogens [4]. Root colonization and rhizosphere competence is thus a critical prerequisite for the successful use of PGPRs as biocontrol and plant growth-promoting agents [5, 6].

The rhizosphere is rich in nutrients due to the accumulation of plant exudates containing amino acids and sugars that provides a rich source of energy and nutrients for colonizing bacteria [7]. This forces the bacterial community to colonize the rhizoplane and rhizosphere [8]. The colonizing capability of PGPR strains has been investigated using GFP labeling and transposon-mediated random mutagenesis approaches, in *Pseudomonas* and *Bacillus* strains, in particular [9, 10]. Bacterial traits such as chemotaxis, motility, attachment, growth, and stress resistance all appear to contribute to the colonization competence of PGPRs [8].

Rhizosphere competence is essentially a process of niche competition between PGPRs and other microbes present in the vicinity of plant roots, in which resource partitioning, competitive exclusion, and co-existence can all play a part. It is reported that PGPRs colonize more efficient in poorer microbial communities than in richer soils [11]. The indigenous rhizosphere microbial community can be influenced by large-scale application of PGPRs in field trials [12]. *Pseudomonas* sp. DSMZ 13134 affects the dominant bacterial community of barley roots in the tested soil system [13], for example. By contrast, as revealed by T-RFLP community fingerprinting, application of *Bacillus amyloliquefaciens* FZB42 did not change the composition of rhizosphere bacterial community in a measurable extent [2]. Similar results were also found for *B. amyloliquefaciens* BNM122 in soybean [14]. Recently, persistence and the effect of FZB42 on the microbial community of lettuce were more deeply analyzed by 454-amplicon sequencing corroborating that inoculation with *B. amyloliquefaciens* has no or only transient minor effects on the microbiota in vicinity of plant roots [15]. A slow decrease in the number of inoculated bacteria was also registered. After five weeks only 55% of the initial number of FZB42 DNA was still traceable within the rhizosphere of lettuce in the field [16]. Unfortunately, till now, the effect of Bacillus PGPR on the non-bacterial members of indigenous plant microbiota has not been analyzed. Moreover, no other representatives of the *B. subtilis* species complex than *B. amyloliquefaciens*, the main source for commercial biofertilizer and biocontrol agents, have been proven for their impact on plant rhizosphere.

*Bacillus subtilis* PTS-394, isolated from the rhizosphere of tomato and fully sequenced in 2014 [17], has been shown to suppress tomato soil-borne diseases caused by *Fusarium oxysporum* and *Ralstonia solanacearum* which is the main obstacle to continuous cropping of tomato *in* greenhouse cultivation [18]. In the present study, the colonization behavior and plant growth-promoting capability of PTS-394 were investigated in laboratory and greenhouse experiments. Additionally, the effect of PTS-394 on the rhizosphere microbial community was studied using Roche 454 pyrosequencing of 16S rRNA and ITS partial sequences. The aim was to improve our understanding of the ecological consequences of microbial inoculants and of rhizosphere microbiota ecology.

## Methods

### Strains and growth conditions

*Bacillus subtilis* PTS-394 and the GFP-tagged strain of *B. subtilis* PTS-394G containing the plasmid pGFP22 were isolated and constructed by our laboratory [19]. The *Bacillus* strains were grown in YPG medium containing 0.5% yeast extract, 0.5% peptone and 0.5% glucose. 5 μg/ml of chloramphenicol was added when necessary for *B. subtilis* PTS-394G.

### The software of Phylogenomic analysis

Phylogenomic analysis of strain PTS-394 was performed taking advantage of availability of the whole genome sequence [17]. JSpeciesWS (http://jspecies.ribohost.com/jspeciesws/) was used to determine ANIb (average nucleotide identity based on BLAST+) and ANIm (average nucleotide identity based on MUMmer) values by pairwise genome comparisons. Correlation indexes of their Tetra-nucleotide signatures (TETRA) were determined by using the JSpeciesWS software (Richter et al., 2015). Electronic DNA-DNA hybridization (dDDH) is useful to mimic the wet-lab DDH and can be used for genome-based species delineation and genome-based subspecies delineation [20, 21] and was applied to finally defined PTS-394 whether belong to *B. subtilis* subsp. *subtilis*. Three formulas are applicable for the calculation: Formula: 1 (HSP length / total length), formula: 2 (identities / HSP length) and formula 3 (identities / total length). Formula 2, which is especially appropriate to analyze draft genomes, was used.

### The colonization and plant growth promotion of PTS-394 on agar plates

*B. subtilis* PTS-394 was shaken at 180 rpm at 28 °C for 36 h, centrifuged at 5000 rpm for 10 min, then the cell pellets were suspended in sterile distilled water and adjusted to an OD_600_ of 0.01, 0.1, 1.0, 1.5, and 3.0 (OD_600_ = 1.0, ~5 × 10^7^ CFU/mL). Tomato (tomato cultivar ‘Moneymaker’) seeds were surface-sterilized using sodium hypochlorite (3%, *v*/v) for 5 min, washed three times with sterile water and soaked in the above cell suspensions at 25 °C for 24 h. Seeds were sown into individual 9 cm diameter MS agar plates [22] at a density of five seeds per plate. Each treatment was performed on 10 plants and was replicated three times. Plates were incubated in a growth chamber with a daytime temperature of 25 °C and a night temperature of 18 °C, 75% humidity and a 12:12 h light: dark cycle with a light intensity of 8000 lux. At 14 days after sowing, plant fresh weight and the amount of PTS-394 on the root surface were measured as previously described [23].

### The colonization and plant growth promotion of PTS-394 in pots

The dynamic colonization of PTS-394 on the tomato rhizoplane and its plant growth-promoting ability were investigated by pot experiments, and the GFP-labeled strain (PTS-394G) was used for colonization analysis. Suspensions of *B. subtilis* PTS-394 and PTS-394G with an OD_600_ = 1.0 were used. In both experiments, tomato seeds were surface-disinfected and sown into nursery soil containing a mixture of vermiculite and organic manure (1:1, *w*/w) for germination. Tomato seedlings at the 4-leaf stage were transplanted into pots filled with a mixture of vermiculite, rice field soil and organic manure (1:2:1, *w*/w). 20 mL of bacterial cell suspension was added to each plant and cultivation was continued in a greenhouse under natural conditions with temperatures ranging from 18 to 30 °C. Each treatment was performed on 30 plants and replicated three times in a completely randomized block design. In the colonization experiment, tomato root samples were collected from three PTS-394G plants at 0, 1, 2, 3, 5, 7, 9, 11, 14, and 21 days after transplantation and the amount of PTS-394 on the root surface was determined, as described previously. Meanwhile, tomato roots were examined at 1, 3, 7 and 14 days using fluorescent microscopy in order to detect colonization with PTS-394G. At 30 days after treatment with PTS-394, the fresh weight of the root and the plant height were measured in order to evaluate the growth-promoting effect of PTS-394.

### Rhizosphere soil collection and DNA extraction

During the pot experiment described above, rhizosphere soil from untreated and PTS-394-treated pot-grown tomato plants was collected from three plants at 1, 3, 7, 9 and 14 days after treatment. Rhizosphere soil total DNA was extracted using the PowerSoil DNA Isolation Kit (MO BIO Laboratories, Inc., Carlsbad, CA, USA) and the quality was estimated using a NanoDrop spectrophotometer (ND-1000, NanoDrop Technologies, Wilmington, USA).

### PCR amplification and Roche 454 pyrosequencing

For analysis of bacteria diversity, partial 16S rRNA genes were amplified using primers 27F (AGAGTTTGATCMTGGCTCAG) and 533R (TTACCGCGGCTGCTGGCAC). Primer pair ITS1 (TCCGTAGGTGAACCTGCGG) and ITS4 (TCCTCCGCTTATTGATATGC) were was used to amplify partial ITS sequences of eukaryotes. Amplifications were performed using the following program: initial denaturation at 95 °C for 2 min, 25 (for 16S rRNA) or 33 (for ITS) cycles of denaturation at 94 °C for 30 s, annealing at 55 °C for 30 s, extension at 72 °C for 30 s, and a final extension at 72 °C for 5 min. Emulsion PCR was performed with the emPCRAmp-Lib L Kit (Roche) and PCR amplicons were pyrosequenced by Shanghai Majorbio Bio-pharm Technology Co. Ltd. (Shanghai, China) using a Roche 454 GS FLX instrument and Titanium reagents. Pyrosequencing generated 84,579 and 157,116 raw ITS and 16S rDNA reads, respectively.

### Processing of 454 sequencing data

Sequences were assigned to different samples according to sample-specific barcodes and processed using QIIME (version 1.17). Data were selected based on the following criteria: (i) an almost perfect match with barcode and primers; (ii) a length of at least 200 nucleotides (barcodes and primers excluded); (iii) an average quality score of >25, with no ambiguous bases or homopolymers longer than six bp, and without any primer mismatches. After this procedure, 67,520 and 140,099 high-quality sequences were obtained for ITS and 16S rDNA reads, respectively. The number of clean reads per samples are listed in Additional file 1: Table S1 and Additional file 2: Table S2.

Operational Taxonomic Units (OTUs) were clustered using a 97% similarity cutoff by UPARSE (version 7.1 http://drive5.com/uparse/) and chimeric sequences were identified and removed using UCHIME. The phylogenetic affiliation of each 16S rRNA gene and ITS sequence were analyzed by RDP Classifier (http://rdp.cme.msu.edu/) against the SILVA (SSU111) 16S/18S rRNA database. Taxonomic assignment from phylum level to strain level based on hits, and these were used to plot abundance graphs.

### Statistical analysis

Plant fresh weight, height and number of colonizing bacteria were analyzed using Microsoft Excel. Calculation of variance (ANOVA) and mean comparison between treatments was carried out based on the Tukey’s test at the 0.05 probability level using SPSS version 19 (IBM Corporation, New York, USA). Principal component analysis (PCA) was performed to analyze the effect of PTS-394 and sampling time on the microbiota community at the OTU level using the R-forge community ecology package (vegan 2.0 was used to generate the PCA Figures).

## Results

### Phylogenomics of *B. subtilis* PTS-394

Phylogenomic analysis of strain PTS-394 was performed taking advantage of availability of the whole draft genome sequence. ANIb and ANIm values in comparison to the *B. subtilis* type strain 168 were determined by using the JSpecies program package. In order to finally decide, whether PTS-394 belong to *B. subtilis* subsp. *subtilis*, electronic DNA-DNA hybridization (dDDH) was applied. For calculating dDDH three different formulas can be applied (see Methods), but only results obtained with the recommended formula 2 were used in our analysis. The nearest neighbors of PTS-394 found by using different phylogenomic analyses were representatives of *B. subtilis* subsp. *subtilis* including the type strain *B. subtilis* 168 (Table 1). Tetra results (tetranucleotide signature correlation index) determined for PTS-394 when compared with *B. subtilis* subsp. *subtilis* 168(T) were in subspecies range (>0,999). The same comparison revealed ANI values far above the recommended species threshold of 0.96% [24] and dDDH values clearly exceeding subspecies delineation (>79%, [21]). By contrast, phylogenomic comparison with other members of the *B. subtilis* species complex including other subspecies of *B. subtilis*, *B. tequilensis*, *B. vallismortis, B. atrophaeus, B.amyloliquefaciens, B. velezensis, B. methylotrophicus, and B. siamensis* did yield values sufficient for species delineation (Table 1). We conclude that strain PTS-394 is a representative of the plant-associated taxon *B. subtilis* subsp. *subtilis.*

**Table 1:** Taxonomic relationship of *B. subtilis* PTS-394 based on whole genome analysis

query	accession	Z-score	ANIb	ANIm	dDDH (formula 2)	Probability		G + C
						>70%	>79%	
*Bacillus subtilis* PTS-394	AWXG00000000							
**reference**								
*Bacillus subtilis subtilis* ASM74047v1	JPVW01000000	**0.99976**	**98.56**	**98.85**	**90.10% [87.8–92.1%]**	**95.85%**	**65.49%**	0.00%
*Bacillus subtilis* QH-1	AZQS00000000	**0.99976**	**98.59**	**98.95**	**91.10% [88.9–92.9%]**	**96.15%**	**66.94%**	0.02%
*Bacillus subtilis subtilis* 168(T)	NC_000964.3	**0.99929**	**98.46**	**98.85**	**90.10% [87.8–92%]**	**95.84%**	**65.47%**	0.18%
*Bacillus subtilis subtilis* MP9	APMW00000000.1	**0.99969**	**98.41**	**98.62**	**87.20% [84.7–89.4%]**	**94.76%**	**60.77%**	0.09%
*Bacillus subtilis* B4143	JXLQ00000000	**0.99969**	**98.38**	**98.65**	**88.00% [85.5–90.1%]**	**95.06%**	**61.99%**	0.03%
*Bacillus subtilis subtilis* RO-NN-1	CP002906.1	**0.99953**	**97.84**	**98.07**	**82.90% [80–85.4%]**	**92.55%**	**53.1%**	0.17%
*Bacillus subtilis spizizenii* W23	NC_014479.1	0.99895	92.59	92.87	49.30% [46.7–52%]	17.32%	3.61%	0.19%
*Bacillus subtilis spizizenii* ATCC 6633	ADGS00000000	0.99901	92.49	92.87	49.20% [46.6–51.8%]	17%	3.54%	0.13%
*B.subtilis inaquosorum* KCTC13429(T)	AMXN00000000	0.99832	92.37	93.05	49.90% [47.3–52.6%]	18.92%	3.93%	0.01%
*Bacillus tequilensis* KCTC 13622(T)	AYTO00000000	0.99741	91.29	91.83	44.90% [42.3–47.4%]	8%	1.76%	0.16%
*Bacillus vallismortis* DV1-F-3	AFSH00000000	0.99729	90.47	91.10	42.50% [40–45%]	4.85%	1.14%	0.06%
*Bacillus mojavensis* KCTC 3706(T)	AYTL00000000	0.99748	86.81	87.58	32.40% [30–34.9%]	0.27%	0.1%	0.03%
*Bacillus mojavensis* RRC 101	ASJT00000000	0.99731	86.74	87.64	32.40% [30–34.9%]	0.27%	0.1%	0.03%
*Bacillus atrophaeus* 1942	CP002207.1	0.98582	79.19	83.88	22.00% [19.8–24.5%]	0%	0%	0.48%
*Bacillus amyloliquefaciens* DSM 7(T)	FN597644.1	0.95303	76.39	83.88	20.40% [18.2–22.8%]	0%	0%	2.39%
*Bacillus amyloliquefaciens* UCMB-5036	NC_020410.1	0.94889	76.33	84.19	20.50% [18.3–22.9%]	0%	0%	2.9%
*Bacillus amyloliquefaciens* UCMB-5113	NC_022081.1	0.94919	76.32	84.12	20.50% [18.2–22.9%]	0%	0%	3.01%
*Bacillus amyloliquefaciens* FZB42(T)	NC_009725.1	0.9508	76.29	84.15	20.50% [18.3–22.9%]	0%	0%	2.78%
*Bacillus amyloliquefaciens* UCMB-5033	NC_022075.1	0.95049	76.26	84.09	20.40% [18.2–22.8%]	0%	0%	2.49%
*Bacillus methylotrophicus* SK19.001	AOFO00000000	0.95157	76.21	84.25	20.40% [18.1–22.8%]	0%	0%	2.47%
*Bacillus methylotrophicus* JS25R	CP009679.1	0.9523	76.16	84.21	20.40% [18.2–22.8%]	0%	0%	2.71%
*Bacillus siamensis* KCTC 13613(T)	AJVF00000000	0.95296	76.16	84.39	20.60% [18.3–23%]	0%	0%	2.64%

Thresholds supporting subspecies delineation (intraspecific Tetra-nucleotide signature correlation index (Z-score): > 0.999, ANIb and ANIm: >98%, and dDDH: >79%) are indicated in bold letters

### PTS-394 is able to colonize roots and to promote growth of tomato plants

**Fig. 1:**
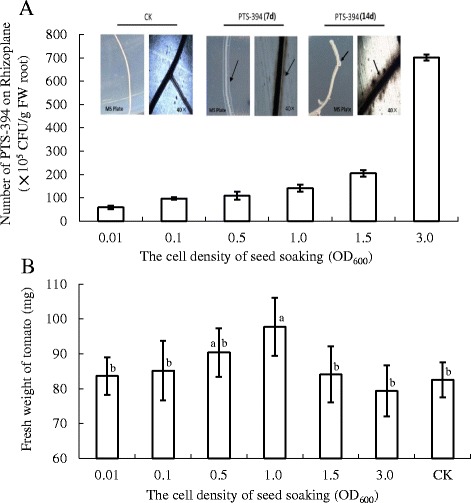
Effect of *B. subtilis* PTS-394 on root colonization and tomato growth on MS agar. **a**, PTS-394 cell number (CFU) detected on the tomato rhizoplane after treatment with suspensions of different cell densities (histogram), and pattern of colonization by PTS-394 (arrows indicate the PTS-394 cells around the tomato root). **b**, Growth promotion effect of PTS-394: Fresh weight of tomato seedlings after soaking with suspensions of different cell densities (histogram). Different letters indicate significant difference at the 0.05 level by Duncan’s new multiple range test

The effect of *B. subtilis* PTS-394 on tomato growth and root colonization was evaluated using MS agar plates. After 7 and 14 days growth, PTS-394 cells became clearly visible around the tomato primary root (Fig. 1a). Density of PTS-394 on the tomato rhizoplane ranged from 10^6^ to 10^7^ CFU per gram of fresh root, and was found enhanced with increasing cell density of the soaking suspension (Fig. 1a). These results indicated successful root colonization by PTS-394, which is a key requirement for biocontrol action and plant growth-promoting activity. Seedling fresh weight data showed that PTS-394 exhibited impressive growth-promoting activity at a cell density (OD_600_) of 0.5 and 1.0 in the soaking suspension, yielding 1.08 × 10^7^ and 1.4 × 10^7^ CFU/g of fresh root on the tomato rhizoplane, respectively (Fig. 1b). These treatments increased plant growth by 9.46% and 18.36%, respectively, compared to controls. However, tomato growth was slightly inhibited when the PTS-394 cell number exceeded 7 × 10^7^ CFU/g of fresh root, suggesting that too high cell numbers at rhizoplane might negatively affect plant growth by that bacterium.

### Evaluation of root colonization and tomato growth promotion by PTS-394 in pot experiments

**Fig. 2:**
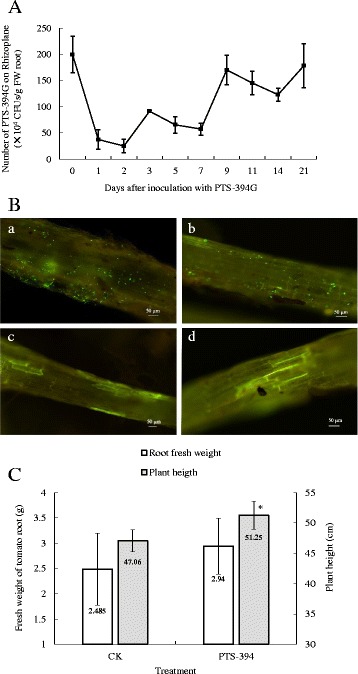
Colonization dynamics and growth promotion of *Bacillus subtilis* PTS-394 in tomato pot experiments. **a**, Bacterial cell counts on tomato plant root surfaces over time following inoculation with *B. subtilis* PTS-394G. **b**, Fluorescent microscopy of tomato roots colonized by PTS-394G. Root samples were collected at 1 (*a*), 3 (*b*), 7 (*c*) and 14 (*d*) days post-treatment. **c**, Effect of PTS-394 on growth promotion as measured by root fresh weight and plant height. Error bars indicate the standard deviation calculated from three independent samples. The asterisk indicates a significant difference at the 0.05 level by Duncan’s new multiple range test

Pot experiments were performed to investigate the colonization capability of PTS-394G at different time intervals. Initially, rate of root colonization decreased sharply before steadily increasing over time (Fig. 2a). Almost 2 × 10^6^ CFU per gram of root were detected immediately after inoculation; 48 h after inoculation this number dropped to 3.7 × 10^5^ CFU/g root. During next days PTS-394G cell number increased steadily and reached 1.7 × 10^6^ CFU/g root nine days after inoculation. Between days 9–21, the PTS-394G cell number ranged between 1 × 10^6^ to 2 × 10^6^ CFU/g root, and fluorescent microscopy revealed that colonization occurred on the root surface, where PTS-394 cells formed micro-colonies or biofilms 7 days after treatment (Fig. 2b). The average plant height and root weight were 51.25 cm and 2.94 g, respectively, after treatment with PTS-394, which represented an increase of 8.90% and 18.30% compared with untreated control plants (Fig. 2c).

### Impact of PTS-394 on bacterial members of the rhizosphere microbiota

Determination of the composition of the bacterial community in vicinity of plant roots was performed by metagenome sequencing (see Methods). A total of 157,116 raw 16S rRNA sequences were obtained from 10 soil samples collected at five different time intervals from PTS-394-treated and untreated plants. The number of clean sequences per sample ranged from 11,773 to 15,879, with an average of 14,010. At 97% sequence similarity, these clean sequences represented a total of 19,231 OTUs by a two-stage clustering (TSC) algorithm and ranged from 4054 to 5343. The Shannon index of diversity was determined for all samples, and ranged from 7.41 to 7.94. The Good’s Coverage per sample ranged from 0.77 to 0.83. The statistical indexes and richness estimates per sample are summarized in Additional file 1: Table S1. Global Alignment for Sequence Taxonomy (GAST) was used for taxonomic assignment of 16S rRNA sequences, and 31 phyla were identified by pooling sequences from all samples, with nineteen core phyla present in all ten samples (Table 2). The three most abundant core phyla were *Proteobacteria*, *Bacteroidetes* and *Actinobacteria*, which were present in all samples, except the one collected on the first day after treatment with PTS-394.

**Table 2:** Relative abundance of nineteen bacterial core phyla present in rhizosphere bacterial community determined by 16S sequencing

Core phylum	Relative abundance (%) in Control treatment					Relative abundance (%) in PTS-394 treatment				
	1 d	3 d	7 d	9 d	14 d	1 d	3 d	7 d	9 d	14 d
*Proteobacteria*	37.94	38.43	40.11	37.13	41.58	36.56	39.18	38.29	41.37	39.22
*Bacteroidetes*	13.85	13.36	12.24	14.26	11.19	11.48	12.27	11.30	11.56	12.45
*Actinobacteria*	9.70	9.43	9.73	11.96	10.67	7.92	7.84	11.20	8.85	8.52
*Chloroflexi*	8.08	8.36	8.42	7.72	6.01	5.96	6.46	7.82	6.54	7.97
*Acidobacteria*	5.70	7.17	6.77	6.79	5.77	5.89	7.65	6.19	7.10	6.27
*Gemmatimonadetes*	6.12	5.28	5.38	4.37	5.36	5.83	6.01	6.12	6.59	6.29
*Firmicutes*	2.52	2.78	2.49	2.81	3.92	13.78	6.68	3.85	3.58	3.29
*Planctomycetes*	5.80	5.21	5.18	4.91	3.93	3.58	4.25	4.66	4.22	3.05
*Cyanobacteria*	0.58	0.50	0.44	0.87	0.86	0.45	0.47	0.55	0.64	1.18
*Verrucomicrobia*	0.39	0.49	0.49	1.06	0.84	0.46	0.40	0.50	0.59	0.56
*Nitrospirae*	0.50	0.83	0.51	0.72	0.47	0.43	0.58	0.42	0.61	0.48
*Chlorobia*	0.48	0.40	0.41	0.28	0.32	0.36	0.40	0.55	0.51	1.09
*Deinococcus-Thermus*	0.35	0.55	0.32	0.30	0.29	0.30	0.35	0.36	0.42	0.33
*Fusobacteria*	0.42	0.53	0.39	0.47	0.21	0.33	0.28	0.24	0.26	0.21
*Armatimonadetes*	0.28	0.29	0.28	0.24	0.43	0.22	0.30	0.37	0.35	0.21
*Fibrobacteres*	0.07	0.08	0.09	0.09	0.18	0.05	0.07	0.09	0.09	0.31
*Elusimicrobia*	0.04	0.05	0.04	0.09	0.15	0.03	0.08	0.06	0.06	0.05
*Thermotogae*	0.05	0.04	0.05	0.04	0.02	0.04	0.01	0.04	0.01	0.03
*Spirochaetes*	0.03	0.03	0.05	0.01	0.02	0.04	0.03	0.03	0.01	0.02

The assay was performed 1, 3, 7, 9, and 14 days after inoculation with PTS-394. Control experiments without PTS-394 were performed at same time points

**Fig. 3:**
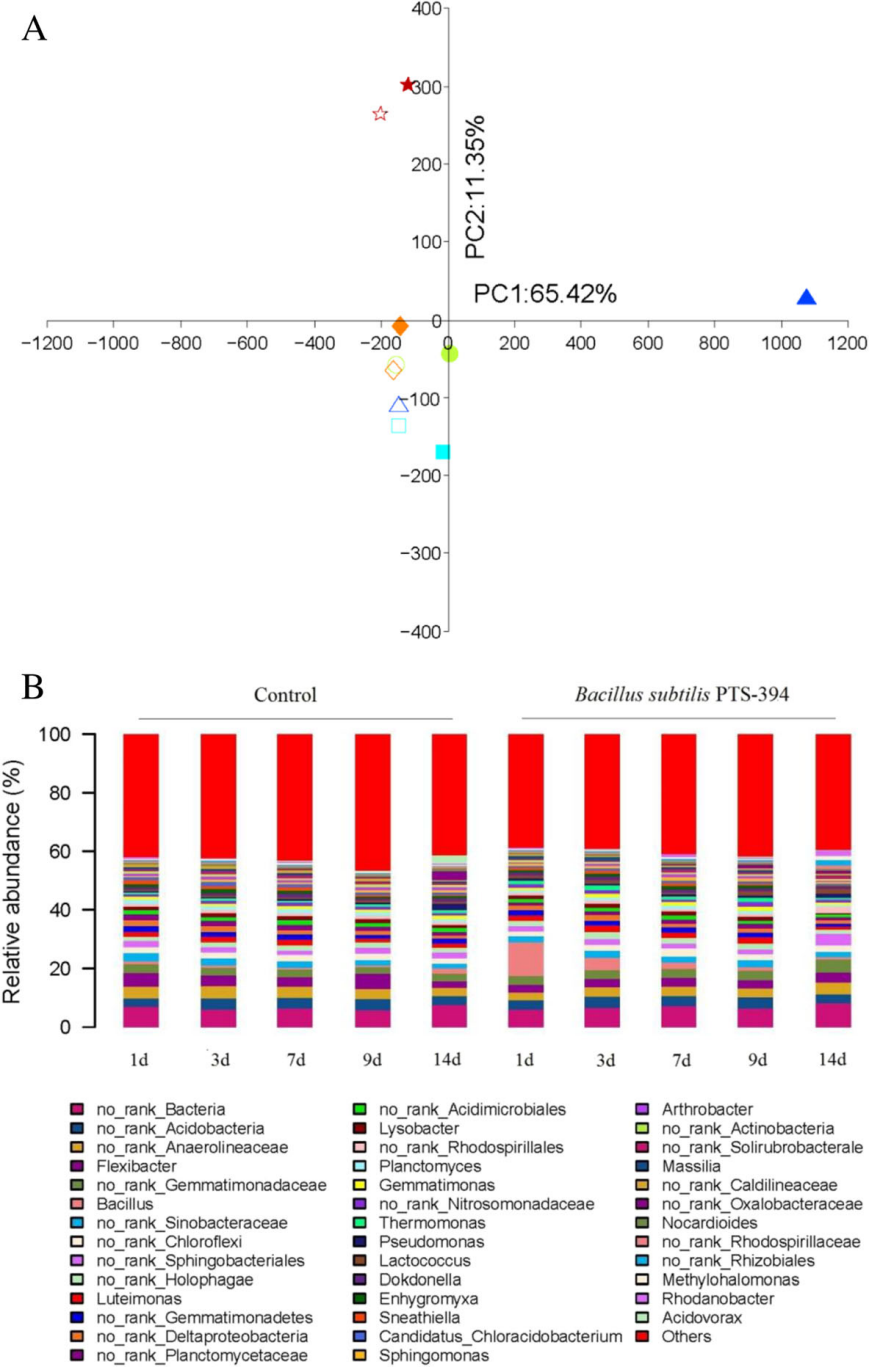
Effect of *Bacillus subtilis* PTS-394 on the rhizosphere bacterial community. **a**, Principal component analysis was performed based on OTUs from16S rRNA partial sequence. The same shape and color represent the same sampling time. △, □, ○, ◇, ☆ represent 1, 3, 7, 9 and 14 days after transplantation. Filled data points represent plants treated with PTS-394 and open data points represent control plants. **b**, relative abundance of 40 core groups (genera) present in the rhizosphere bacterial community

Principal component analysis (PCA) was used to analyze the effect of PTS-394 and sampling time on the structure of the bacterial community in the rhizosphere based on OTUs. PC1 and PC2 together accounted for more than 75% of the variation (Fig. 3a). There was a clear shift in bacterial community between 1 and 14 days after inoculation with PTS-394. Treated or untreated samples collected on day 1 were distinct from each other and from the other time points, however control and treated samples could not be distinguished at later stages. These results indicated that (i) changes in the bacterial community profile becomes visible soon after inoculation with *B. subtilis*, and (ii) application of PTS-394 affected only transiently the root bacteriome. As expected, a sudden rise of *Firmicutes* was registered due to the large quantity of *B. subtilis* PTS-394 cells applied. However, in course of the experiment the number of *Firmicutes* was steadily decreasing and became similar with the control 14 days after inoculation (Table 2).

BLAST analysis revealed that the relative abundance of 40 different groups (based on genus level under inclusion of some uncertain groups) was temporarily affected after inoculation (Fig.3b). A total of 19 groups were altered by PTS-394 treatment, occurrence of 10 groups was enhanced, including *Thermomona*s, *Pseudomonas*, *Lactococcus* and *Rhodanobacter*, while nine groups were suppressed, including *Flexibacter, Planctomyces* and *Sneathiella*. However, as in *Firmicutes* (see above), these groups were only temporarily affected and 14 days after inoculation no significant differences to the control were registered. The relative abundance and variation in the composition of these 19 groups is presented in Additional file 3: Figure S1.

### Impact of PTS-394 on eukaryotic members of the rhizosphere microbiota

Presence of eukaryotic groups in vicinity of plant roots was determined by using 18S rRNA sequences in a similar way as described for bacteria. In total, 84,579 raw ITS sequences were obtained and the number of clean sequences per sample varied ranged from3, 897 to 8402, with an average of 6752. A total of 1491 Operational Taxonomic Units (OTUs) were obtained at a distance of 0.03 using the TSC algorithm. The Shannon index of diversity was determined for 10 samples, and ranged from 0.28 to 4.15. The Good’s coverage per sample ranged from 0.96 to 0.99. Although there was some fluctuation of reads over time, analysis of these data corroborated their credibility. The statistical indexes and richness estimates of per sample are summarized in Additional file 2: Table S2. The eukaryotic OTUs could be arranged into three kingdoms (*Fungi*, *Metazoa* and *Viridiplantae*) along with the *no rank Eukaryota* group, a group of *unclassified Eukaryota* and a group of *unclassified* organisms. Altogether, thirteen phyla were identified. Seven core phyla were detected in every sample. The most abundant phyla were *Streptophyta*, *Chlorophyta Ascomycota* and *Basidiomycota* (Table 3). After the application of PTS-394, the composition of *Fungi*, *Viridiplantae*, the *no rank Eukaryota* group and the *unclassified* group were altered, compared to the untreated control. The relative abundance of *Fungi*, of the *no rank Eukaryota* group, and of the group of *unclassified* organisms was lower than that of the control at the same sample time. However, the relative abundance of *Viridiplantae* (*Solanoideae*) was found higher than in the control.

**Table 3:** Relative abundance of core kingdoms/phylum present in rhizosphere eukaryota community determined by ITS sequencing

Core Kingdom	Relative abundance (%) in Control treatment					Relative abundance (%) in PTS-394 treatment				
	1 d	3 d	7 d	9 d	14 d	1 d	3 d	7 d	9 d	14 d
*Fungi*	16.24	20.54	5.64	36.52	1.11	17.22	12.10	5.91	9.33	1.53
*Viridiplantae*	29.79	24.93	81.81	39.59	97.76	58.22	79.23	92.29	81.40	97.37
*Metazoa*	0.39	0.62	0.05	0.98	0.17	0.63	0.15	0.43	1.09	0.14
*no_rank_Eukaryota group*	20.37	21.83	6.21	20.69	0.20	22.27	7.93	1.08	7.04	0.94
*unclassified_Eukaryota group*	0.18	0.20	0.08	0.16	0.00	0.06	0.04	0.03	0.01	0.01
*Unclassified group*	33.03	31.87	6.21	2.05	0.76	1.59	0.55	0.27	1.12	0.00
Core phylum										
*Streptophyta*	19.87	17.29	74.15	30.06	97.43	18.17	64.02	90.46	70.60	97.32
*Chlorophyta*	9.91	7.59	7.64	9.39	0.33	40.04	15.20	1.83	10.80	0.05
*Ascomycota*	2.17	3.98	1.30	8.42	0.13	2.26	1.44	0.59	0.67	0.63
*Basidiomycota*	1.94	1.83	0.83	2.10	0.17	1.60	4.36	0.94	1.13	0.16
*Porifera*	0.37	0.49	0.05	0.79	0.06	0.19	0.12	0.42	0.78	0.01
*Chytridiomycota*	0.18	0.42	0.20	0.58	0.03	0.40	0.19	0.11	0.23	0.07
*Nematoda*	0.03	0.09	0.00	0.15	0.12	0.41	0.03	0.01	0.11	0.10
*no_rank_Eukaryota group*	20.37	21.82	6.19	20.68	0.20	22.26	7.93	1.05	7.04	0.94
*no_rank_Fungi group*	11.19	12.94	3.13	18.86	0.67	12.44	5.75	4.18	6.99	0.66
*unclassified_Eukaryota group*	0.18	0.20	0.08	0.16	0.00	0.06	0.04	0.03	0.01	0.01
*unclassified_Fungi group*	0.71	1.30	0.18	6.48	0.10	0.44	0.28	0.08	0.14	0.01

The assay was performed 1, 3, 7, 9, and 14 days after inoculation with PTS-394, Control experiments without PTS-394 were performed at same time points

**Fig. 4:**
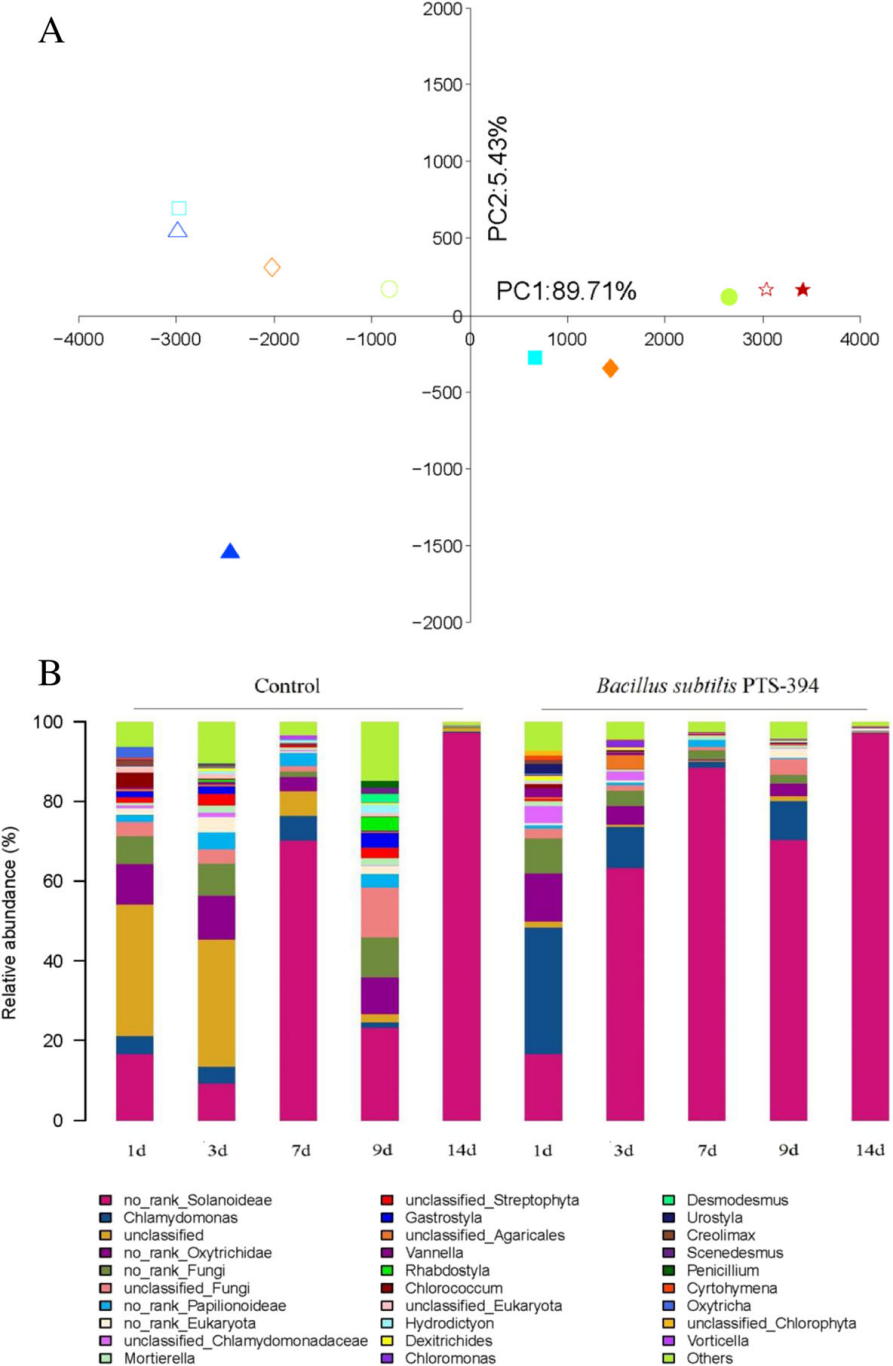
Effect of *B. subtilis* PTS-394 on the rhizosphere Eukaryota community. **a**, Principal component analysis was performed based on OTUs from ITS partial sequences. The same shape and color indicate the same sampling time. △, □, ○, ◇, ☆ represent 1, 3, 7, 9 and 14 days after transplantation. Filled data circles represent plants treated with PTS-394 and open circles represent control plants. **b**, relative abundance of 29 core groups (genera) present in the rhizosphere Eukaryota community

All OTUs were subjected to PCA analysis and PC1 and PC2 together accounted for more than 90% of the variation (Fig. 4a). A significant temporal change in the community profile of Eukaryota is visible in the biplot. Profiles of control and PTS-394-treated samples collected at identical time points revealed slight differences from day 1 until day 9 after treatment with PTS-394, but could not be distinguished 14 days after inoculation. These results are in accordance to those found for the rhizosphere bacteriome, and indicate that (i) PTS-394 had a transient influence on composition of the eukaryotic members of the rhizosphere microbiome, but (ii) the community recovered to its original state 14 days after treatment. BLAST analysis revealed that the relative abundance of 29 groups (based on the eukaryota genus level and including some uncertain groups) was transiently affected after inoculation with PTS-394 (Fig. 4b). A total of 19 groups were altered by PTS-394 treatment; five groups were enhanced, including *Solanoideae, Chlamydomonas* and *Chloromonas*, and 14 groups were decreased. The variation in these 19 groups is presented in Additional file 4: Figure S2.

**Fig. 5:**
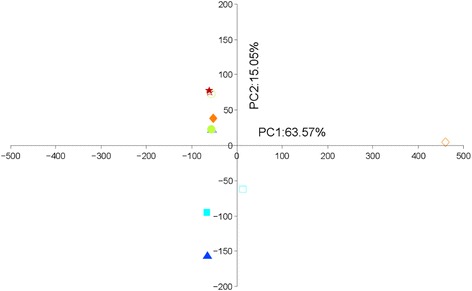
Effect of *B. subtilis* PTS-394 on the rhizosphere fungi community. Principal component analysis was performed based on OTUs from fungi ITS partial sequences. The same shape and color represent the same sampling time. △, □, ○, ◇, ☆ represent 1, 3, 7, 9 and 14 days after transplantation. Filled circles represent plants treated with PTS-394 and open circles represent control plants

PCA on OTUs classified as fungi revealed a temporal variation in the fungal community in dependence of PTS-394 inoculation (Fig. 5). The Control and the PTS-394-treated samples were found different during the first four time points, suggesting that PTS-394 inoculation affects the fungal community transiently, similar to its effect on the bacterial community. Sequence homology and analysis of the relative abundance of OTUs showed that abundance of fungi was generally suppressed during plant development, however by PTS-394 this process was slightly enhanced (Table 3). As expected, the relative abundance of *Fusarium oxysporum* was inhibited after treatment with PTS-394 indicating antagonistic activity of PTS-394 against *F. oxysporum* (Additional file 5: Figure S3).

## Discussion

Plant growth promotion and biocontrol activities are important features of commercial agents used in sustainable agriculture. To date members of the genus *Bacillus* are preferred for preparing bioformulations with beneficial impact on plant growth and health [1]. Especially, representatives of the *B. subtilis* species complex are known for their beneficial action on plants, especially *B. amyloliquefaciens* and *B. subtilis* [25]. These *Bacilli* stimulate plant growth: (1) directly, by increasing nutrients through the production of phytohormones, siderophores, organic acids involved in P-solubilisation and/or fixation of nitrogen [26]; and (2) indirectly, by producing antagonistic substances or by inducing the plant resistance against pathogens [7]. Here, we found that plant root-colonizing *B. subtilis* subsp. *subtilis* PTS-394G is able to persist on roots and to promote growth of tomato plants. Moreover, the plant pathogen *Fusarium oxysporum* was suppressed in presence of PTS-394. Laboratory MS plate experiments showed that the amount of PTS-394G on the tomato rhizoplane was approximately 10^7^ CFU per gram of fresh root, while the cell number was 10^6^ CFU in pot experiments. This suggests that sterile environment favors root colonization of PTS-394, which is consistent with previous reports [11, 27]. It is interesting to note that the plant growth promoting effect exerted by PTS-394 was dependent on the amount of *B. subtilis* cells used for inoculating tomato plants. Whilst cell numbers of 1.08 × 10^7^ and 1.4 × 10^7^ CFU/g fresh root supported plant growth, higher cell numbers such as 7 × 10^7^ CFU/g fresh root, led to a slight growth inhibition. Without genetic experiments it remains questionable whether this different pattern of growth stimulation could be explained by a concentration dependent effect of the phytohormone indole-3-acetic acid (IAA). The PGPR *B. amyloliquefaciens* FZB42 is able to produce IAA and it is possible that higher concentrations of IAA exerts an inhibitory effect on plant growth [1]. In fact, *ysnE*, a gene encoding a putative IAA acetyltransferase, which is involved in IAA synthesis pathway, was detected in the genome of PTS-394. Our results demonstrate that, besides *B. amyloliquefaciens* and *B. pumilus*, plant-associated representatives of the *B. subtilis* subsp. *subtilis* taxon are promising candidates for developing of efficient biocontrol and biofertilizer agents. In this context it is important to know, whether addition of PTS-394 has an effect on composition of rhizosphere microbial community.

Here, the effects of PTS-394 on the entire rhizosphere microbiota community was investigated by taxonomic profiling of metagenome sequences. In accordance with the results obtained for *B. amyloliquefaciens* FZB42 [2, 15, 16], only a transient effect on composition of the root microbiome was detected after adding the PGPR bacterium. Similarly, independent of its mode of application, applying *B. amyloliquefaciens* BNM122 to soy bean plants did not shift the composition of rhizosphere bacterial community in a measurable extent [14], suggesting that inoculation of crops with different representatives of the genus *Bacillus* has no durable impact on the bacteria living in vicinity of plant roots. By contrast, external addition of Gram-negative PGPR, such as *Pseudomonas* spp. [15, 28], *Enterobacter cowanii* [29], and *Sinorhizobium meliloti* [30] alter composition of root bacterial community in a similar extent as reported for fungal plant pathogens, e.g. *Ralstonia solani* [2].

Whilst the impact of PGPRs on the rhizosphere bacterial community is fairly investigated, relatively little is known about the effect of PGPRs on the overall eukaryotic community [8]. Analysis of 18S ribosomal ITS sequences showed that PTS-394 influences Eukaryota in the rhizosphere microbial community. However, similar to the effects exerted by rhizosphere bacteria, the impact of PTS-394 on Eukaryota was only transient, although longer-lasting than its effect on bacterial community.

The rhizosphere contains amino acids and sugars nutrients due to the accumulation of root exudates that provides a rich source of energy and nutrients for rhizosphere microbiome. This determines composition of the microbiome community [7, 8]. After application of PTS-394, relative abundance of *Fungi*, *Viridiplantae*, the *no rank Eukaryota* group and the *unclassified* group was similar as in the control, but changes in their relative abundance compared to control were registered. Here, a significant increase of *Viridiplantae* relative abundance was observed which might be due to enhanced root development caused by PTS-394 as seen by enhanced number of *streptophyta* counts largely corresponding to tomato cells. *Chlorophyte,* another group of the *Viridiplantae*, the relative abundance was decreased significantly in the PTS-394 treatment, which correspond to the variation trend of main genus *Chlamydomonas*. In addition, PGPR have the ability to secrete several antagonistic compounds, such as antifungal acting lipopeptide antibiotics. Nihorimbere et al. reported that surfactin, iturin and fengycin were detected when *Bacillus amyloliquefaciens* S499 colonized tomato rhizosphere [31]. We found that suspensions of *B. subtilis* PTS-394 contained lipopeptides and polyketides. Whilst PTS-394 colonizes plant root, lipopeptides and polyketides might be secreted by the strain into the environment. These secondary compounds will either directly inhibit members of the microbiome community or stimulate the ISR response in plants. The suppressing effect of PTS-394 on plant pathogen *Fusarium oxysporum* in the rhizosphere is a typical example (Additional file 5: Figure S3). We assume that the transient effect of *Bacillus subtilis* PTS-394 on composition of the rhizosphere microbiome is caused by a sudden rise of *Bacillus* cells after inoculation, which is soon compensated by the indigenous microbial community. In case of eukaryota this restoration process is somewhat delayed due to their longer generation time compared to bacteria.

## Conclusions

The impact of PGPRs on the overall rhizosphere community should be considered as important criteria when assessing their suitability for commercial development. Here we found that plant-growth-promoting and biocontrol *Bacillus subtilis* subsp. *subtilis* PTS-394 is not aggressive towards the indigenous tomato rhizosphere microbiota including their eukaryotic representatives, and has only a transient impact on the composition of the community. This, together with its beneficial effect on plant growth and health, makes PTS-394 to a promising candidate for developing a successful PGPR-based bioagent.

## Additional files

Additional file 1: Table S1. Statistical indexes and richness estimates of the rhizosphere bacterial sequence data. (DOCX 29 kb)

Additional file 2: Table S2. Statistical indexes and richness estimates of the rhizosphere eukaryote sequence data. (DOCX 29 kb)

Additional file 3: Figure S1. Variation trends in the abundance of 19 bacterial genera or groups following treatment with *Bacillus subtilis* PTS-394 (part 1 includes 10 groups stimulated by PTS-394, part 2 includes nine groups suppressed by PTS-394). (DOC 7802 kb)

Additional file 4: Figure S2. Variation trends in the abundance of 19 *Eukaryota* genera or groups following treatment with *Bacillus subtilis* PTS-394 (part 1 includes five groups stimulated by PTS-394, part 2 includes 14 groups suppressed by PTS-394). (DOC 6196 kb)

Additional file 5: Figure S3. The variation trends of Relative abundance of *Fusarium oxsysporum* following treatment with Bacillus subtilis PTS-394 (DOC 84 kb)

## Abbreviations

ANIb: Average nucleotide identity based
ANIm: Average nucleotide identity based
dDDH: Electronic DNA-DNA hybridization.
OTUs: Operational taxonomic units
PCA: Principal component analysis
PGPR: Plant growth promoting rhizobacteria
TETRA: Tetra-nucleotide signatures
YPG medium: Yeast peptone glucose medium
