# Supplementary material for: Addition of plant-growth-promoting Bacillus subtilis PTS-394 on tomato rhizosphere has no durable impact on composition of root microbiome
Source: BMC Microbiol. 2017 Jun 5;17:131. doi: 10.1186/s12866-017-1039-x (PMC5460418; doi:10.1186/s12866-017-1039-x)
Supplement: Supplementary file 1 — Statistical indexes and richness estimates of the rhizosphere bacterial sequence data. (DOCX 29 kb) [file 12866_2017_1039_MOESM1_ESM.docx]

Table S1. Statistical indexes and richness estimates of the rhizosphere bacterial sequence data

| **Sequence area** | **Sample** | **reads** | **OTUs** | **Alpha-diversity （0.03）** | | | |
| --- | --- | --- | --- | --- | --- | --- | --- |
|  |  |  |  | **Chao1** | **Shannon index** | **Simpson index** | **Good’s Coverage** |
| 16s-533R-27F | Control (1d) | 11773 | 4275 | 9271 | 7.68 | 0.0011 | 0.77 |
|  | Control (3d) | 13810 | 4671 | 9592 | 7.75 | 0.0009 | 0.80 |
|  | Control (7d) | 11120 | 4166 | 8741 | 7.71 | 0.001 | 0.76 |
|  | Control (9d) | 15462 | 5343 | 10669 | 7.94 | 0.0007 | 0.79 |
|  | Control (14d) | 13853 | 4779 | 9634 | 7.79 | 0.0019 | 0.79 |
|  | PTS-394 (1d) | 15879 | 4792 | 9716 | 7.41 | 0.0071 | 0.82 |
|  | PTS-394 (3d) | 15144 | 4656 | 8845 | 7.65 | 0.0014 | 0.82 |
|  | PTS-394 (7d) | 15039 | 4781 | 9910 | 7.73 | 0.001 | 0.81 |
|  | PTS-394 (9d) | 14583 | 4904 | 9695 | 7.79 | 0.001 | 0.80 |
|  | PTS-394 (14d) | 13436 | 4054 | 7671 | 7.45 | 0.0018 | 0.83 |
