# Supplementary material for: Addition of plant-growth-promoting Bacillus subtilis PTS-394 on tomato rhizosphere has no durable impact on composition of root microbiome
Source: BMC Microbiol. 2017 Jun 5;17:131. doi: 10.1186/s12866-017-1039-x (PMC5460418; doi:10.1186/s12866-017-1039-x)
Supplement: Supplementary file 2 — Statistical indexes and richness estimates of the rhizosphere eukaryote sequence data. (DOCX 29 kb) [file 12866_2017_1039_MOESM2_ESM.docx]

Table S2. Statistical indexes and richness estimates of the rhizosphere eukaryote sequence data

| **Sequence area** | **Sample** | **reads** | **OTUs** | **Alpha-diversity（0.03）** | | | |
| --- | --- | --- | --- | --- | --- | --- | --- |
|  |  |  |  | **Chao1** | **Shannon index** | **Simpson index** | **Good’s Coverage** |
| ITS-ITS1-ITS4 | Control (1d) | 3897 | 300 | 503 | 3.45 | 0.12 | 0.96 |
|  | Control (3d) | 8402 | 530 | 929 | 4.15 | 0.055 | 0.97 |
|  | Control (7d) | 4073 | 184 | 287 | 1.7 | 0.49 | 0.98 |
|  | Control (9d) | 6989 | 485 | 716 | 4.13 | 0.066 | 0.97 |
|  | Control (14d) | 7046 | 80 | 179 | 0.28 | 0.93 | 0.99 |
|  | PTS-394 (1d) | 7934 | 470 | 732 | 3.62 | 0.10 | 0.97 |
|  | PTS-394 (3d) | 6992 | 317 | 507 | 2.17 | 0.39 | 0.98 |
|  | PTS-394 (7d) | 7274 | 184 | 315 | 0.89 | 0.77 | 0.98 |
|  | PTS-394 (9d) | 7461 | 279 | 459 | 1.8 | 0.49 | 0.98 |
|  | PTS-394 (14d) | 7452 | 94 | 153 | 0.32 | 0.92 | 0.99 |
