# Supplementary material for: Addition of plant-growth-promoting Bacillus subtilis PTS-394 on tomato rhizosphere has no durable impact on composition of root microbiome
Source: BMC Microbiol. 2017 Jun 5;17:131. doi: 10.1186/s12866-017-1039-x (PMC5460418; doi:10.1186/s12866-017-1039-x)

Fig. S1. Variation trends in the abundance of 19 bacterial genera or groups following treatment with *Bacillus subtilis* PTS-394 (part 1 includes 10 groups stimulated by PTS-394, part 2 includes nine groups suppressed by PTS-394).

**Part 1**


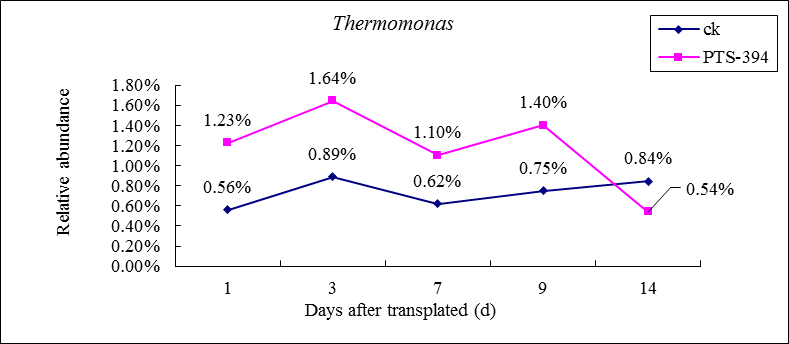

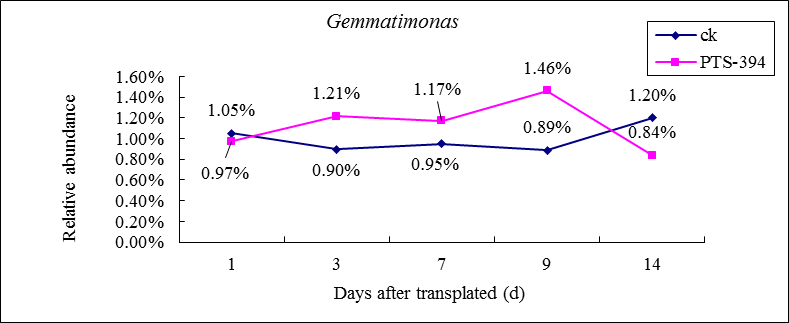


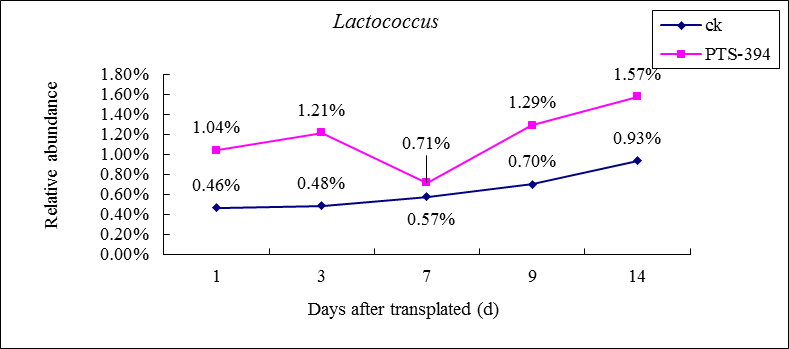

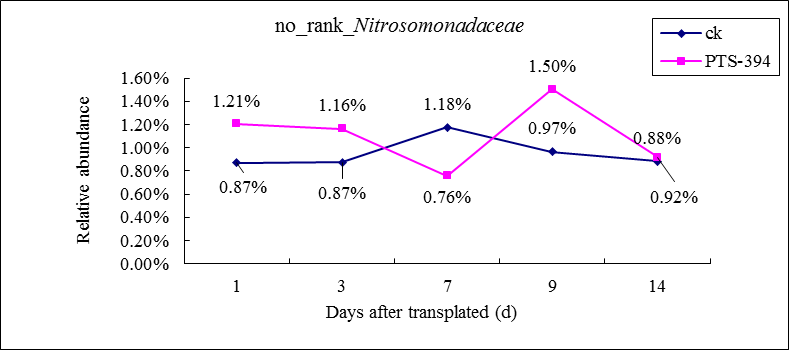


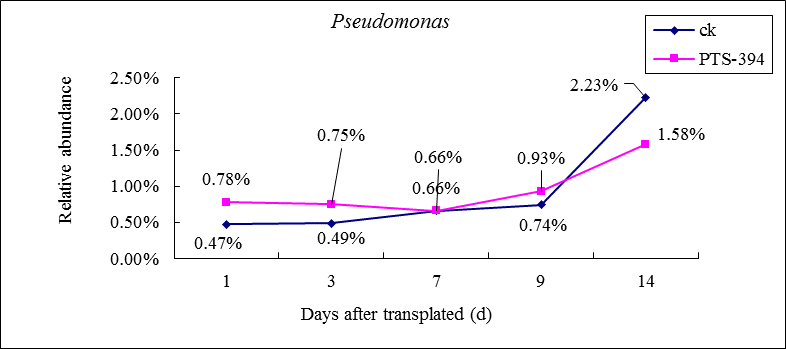


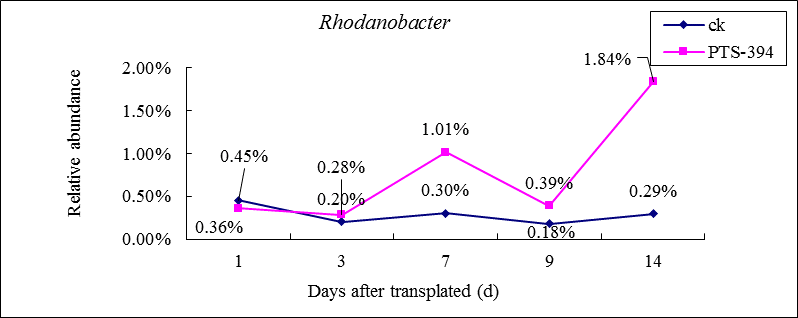


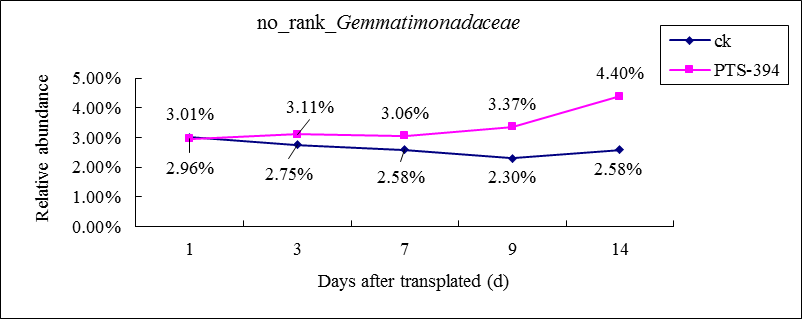


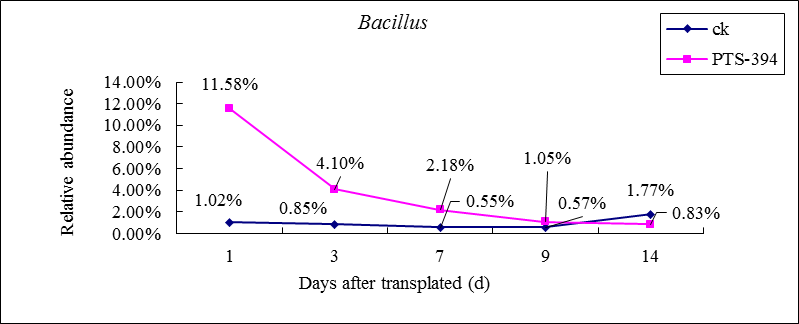


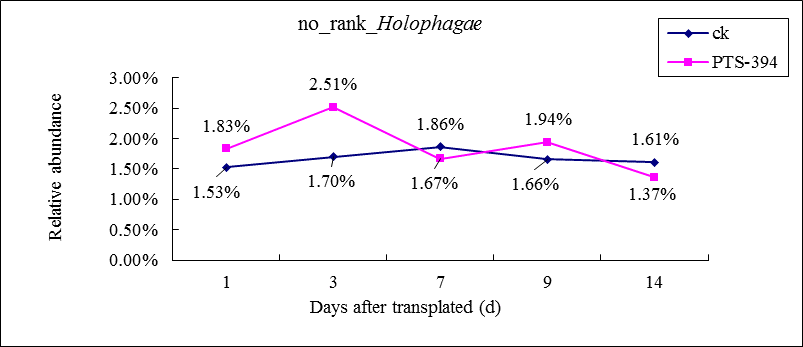


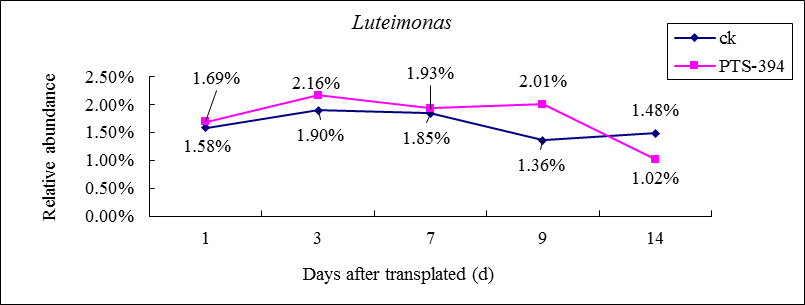


**Part 2**


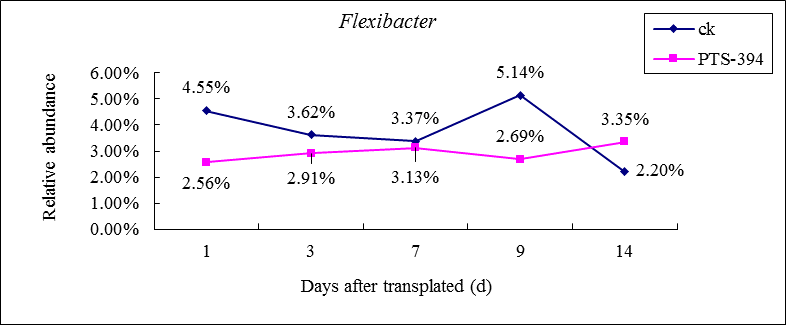

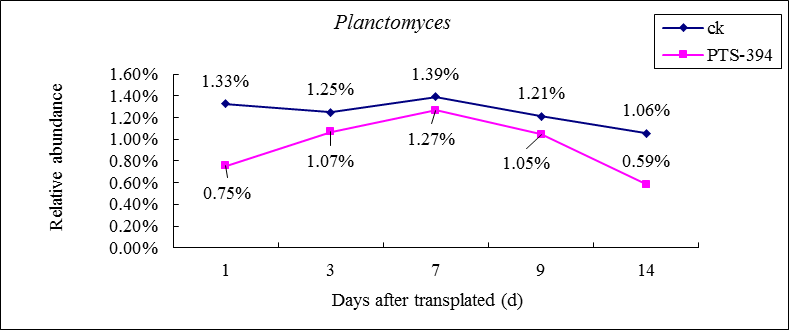

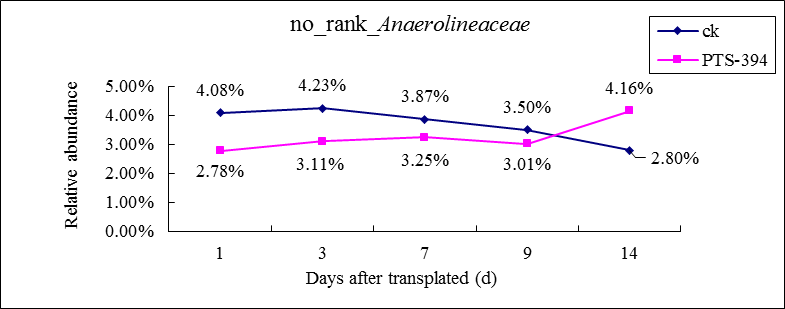


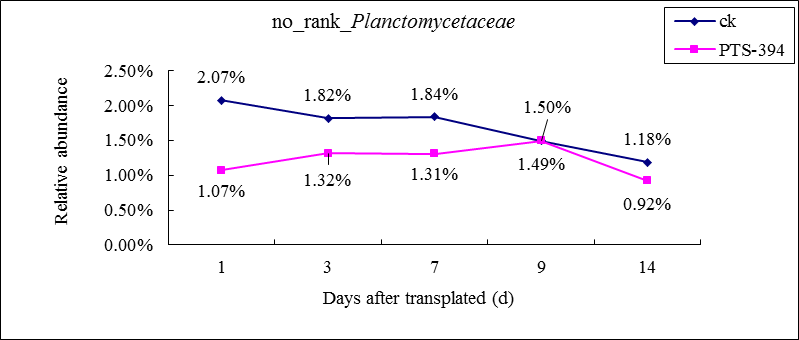


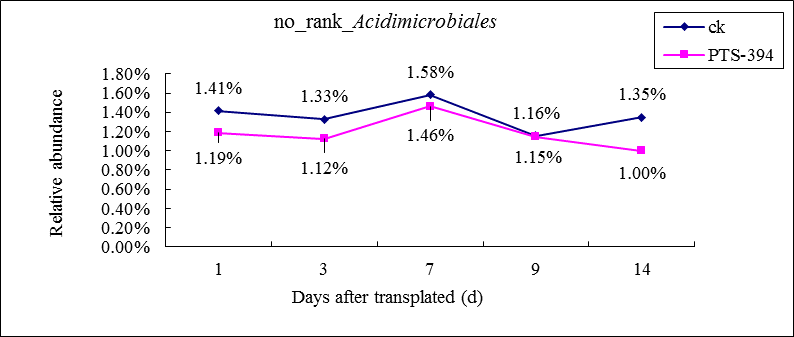


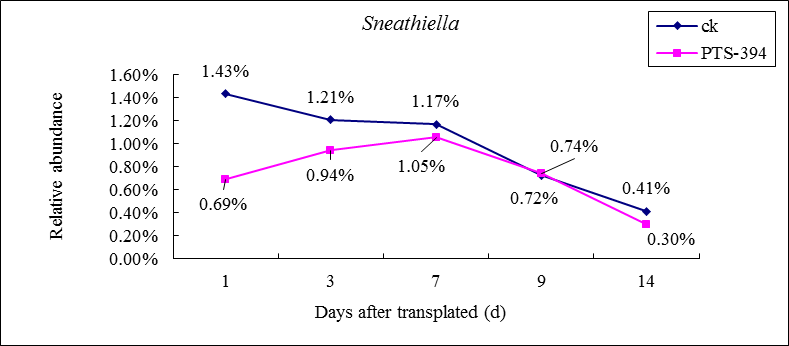


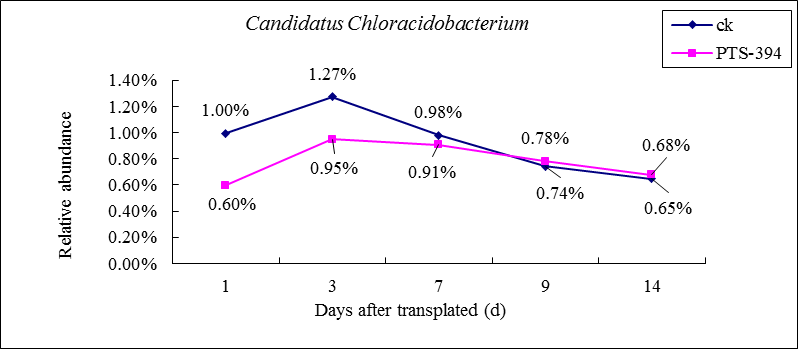


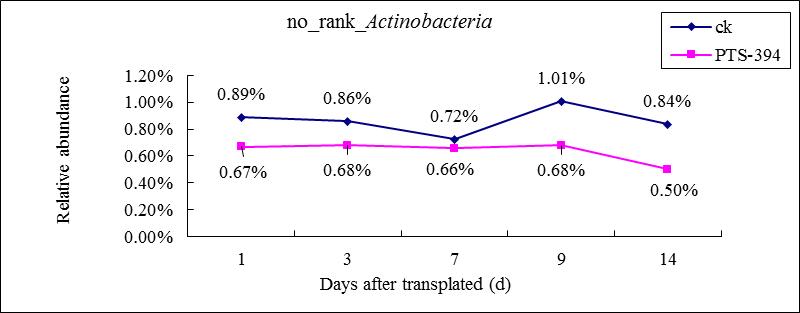


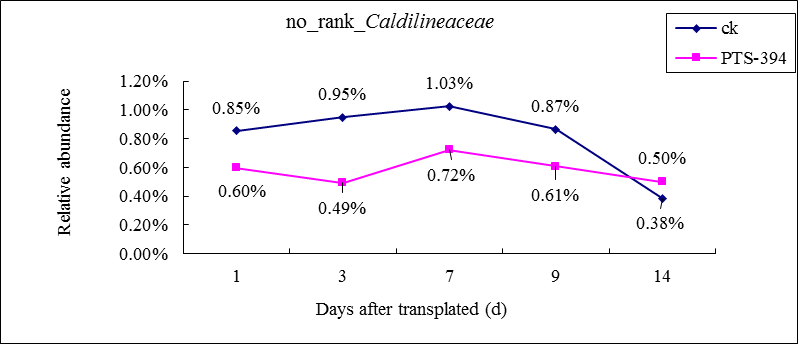

Supplement: Supplementary file 3 — Variation trends in the abundance of 19 bacterial genera or groups following treatment with Bacillus subtilis PTS-394 (part 1 includes 10 groups stimulated by PTS-394, part 2 includes nine groups suppressed by PTS-394). (DOC 7802 kb) [file 12866_2017_1039_MOESM3_ESM.doc]
