# Supplementary material for: Addition of plant-growth-promoting Bacillus subtilis PTS-394 on tomato rhizosphere has no durable impact on composition of root microbiome
Source: BMC Microbiol. 2017 Jun 5;17:131. doi: 10.1186/s12866-017-1039-x (PMC5460418; doi:10.1186/s12866-017-1039-x)

Fig. S3. Variation trends of Relative abundance of *Fusarium oxsysporum* following treatment with *Bacillus subtilis* PTS-394.


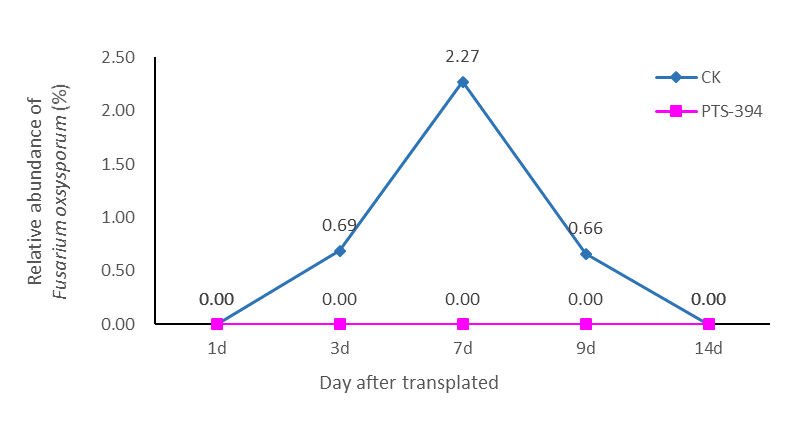

Supplement: Supplementary file 5 — The variation trends of Relative abundance of Fusarium oxsysporum following treatment with Bacillus subtilis PTS-394 (DOC 84 kb) [file 12866_2017_1039_MOESM5_ESM.doc]
